# Study of Probiotic Effects of *Bifidobacterium animalis* subsp. *lactis* BB-12 and *Lactobacillus plantarum* 299v Strains on Biochemical and Morphometric Parameters of Rabbits after Obesity Induction

**DOI:** 10.3390/biology10020131

**Published:** 2021-02-07

**Authors:** Assia Bouaziz, Amira Leila Dib, Nedjoua Lakhdara, Louiza Kadja, Elena Espigares, Elena Moreno, Omar Bouaziz, Mohammed Gagaoua

**Affiliations:** 1 GSPA Research Laboratory, Institut des Sciences Vétérinaires, Université Frères Mentouri Constantine 1, 05 Route de Batna, El-Khroub, Constantine 25000, Algeria; bouaziz.assialp@gmail.com (A.B.); dibamira@hotmail.com (A.L.D.); nedjoua2002@hotmail.com (N.L.); louiza20132014@gmail.com (L.K.); omar.repro@yahoo.fr (O.B.); 2 Department of Preventive Medicine and Public Health, Faculty of pharmacy, University of Granada, Campus Universitario de Cartuja, 18071 Granada, Spain; elespi@ugr.es (E.E.); elmorol@ugr.es (E.M.); 3 Food Quality and Sensory Science Department, Teagasc Food Research Centre, Ashtown, D15 DY05 Dublin 15, Ireland

**Keywords:** rabbits, fatty diet, probiotics, microbiota, health, body weight

## Abstract

**Simple Summary:**

On the basis of the extensive literature, two main strategies have been used to manipulate intestinal microbial composition and selectively stimulate the growth and activity of certain species, these being the administration of either prebiotics or food supplements containing living bacteria such as probiotics. Several animal studies have indicated that certain probiotics, including *Lactobacilli* and *Bifidobacteria*, can suppress body weight gain in rodents, while some probiotics strains have little effect or promote weight gain. The potential anti-obesity effect of probiotics seems to depend on the strains used and the underlying mechanisms, leading to their effects remaining not fully understood. It is in this context that this study was designed to investigate the potential of two probiotics strains, these being *Bifidobacterium animalis* subsp. *lactis* BB-12^®^ and *Lactobacillus plantarum* 299v^®^ in rabbits, whereby obesity and metabolic syndrome was first induced in a first experiment, and the animals were then used in a second experiment to test the hypothesis of probiotics effect on biochemical and morphometric parameters. The model of obesity induced by giving a “cafeteria” diet for 14 weeks in this trial demonstrated a change in the biochemical and morphometric parameters investigated in the ITELV2006 rabbit strain. This study revealed that *B. animalis* subsp. *lactis* BB-12 and *L. plantarum* 299v strains could exert beneficial effects in reducing the incidence of obesity and metabolic syndrome in the ITELV2006 rabbit strain.

**Abstract:**

This study aimed first to develop an experimental model of obesity and metabolic syndrome over 14 weeks using a diet called “cafeteria”, which is a high-fat diet, to evaluate its consequences on the biochemical and morphometric parameters in ITELV2006 strain rabbits. Second, the trial aimed to evaluate the effect of two strains of probiotics, these being *Bifidobacterium animalis* subsp. *lactis* BB-12^®^ and *Lactobacillus plantarum* 299v^®^, on the obesity and MetS induced during the first experiment. Overall, the results of the “cafeteria” diet demonstrated significant changes in numerous biochemical and morphometric parameters, reproducing obesity and the main clinical manifestations of the metabolic syndrome in humans. The administration of the two probiotic strains demonstrated an impact on certain parameters of obesity and induced MetS. This study makes it possible to conclude that probiotics could be useful in the treatment of obesity and metabolic syndrome of rabbits, but in a dependent manner. Furthermore, this study evidenced the importance of selecting specific probiotic strains and dosages to achieve desirable results on rabbits or other species.

## 1. Introduction

In recent decades, the incidence of obesity has increased dramatically and it is becoming a real global epidemic and a major public health concern, with high prevalence worldwide [1]. In 2016, the World Health Organization (WHO) reported that more than 1.9 billion human adults over 18 years of age were overweight; among these, 650 million were obese. In addition, 38.2 million children under 5 years old were estimated in 2019 to be overweight or obese. In Africa, the number of overweight or obese children has increased by almost 24% since 2000.

Multiple environmental and genetic factors are involved in the development of the mechanisms of obesity, mainly caused by a pathological excess of fat mass [2,3,4]. In fact, the increase of fat mass is a result of a chronic disparity in the energy balance and is due to a radical change of diets and habits that is actually characterized by an increased consumption of lipids, responsible consequently for the spectacular emergence of metabolic syndrome disorder related to obesity [5,6,7]. However, it is worthy to note that this metabolic syndrome disorder is a collection of cardiovascular and metabolic alterations such as abdominal obesity, lowered HDL, elevated cholesterol and triglycerides, glucose intolerance and hypertension. Similarly, the diagnostic requires that three of the five criteria mentioned above have to be present [8]. On the other hand, intestinal microbiota is an additional parameter described to be related with obesity and is believed to be one of the onset factors causing the metabolic syndrome [2]. In fact, microbiota represents all the microorganisms living in a specific environment (the gastrointestinal tract), which is composed of more than 100 billion organisms, mainly represented by bacteria [9]. Overall, microbiota improves the ability to extract and store energy from foods [10].

The imbalances in the gut microbiota and the increase in plasma lipopolysaccharides level may also act as inflammatory factors related to the development of glucose intolerance and to body weight gain [2]. According to Tremaroli and Bäckhed [11], the intestinal microbiota has an impact on body weight. This causal relation between intestinal microbiota and obesity was first demonstrated by Turnbaugh et al. [12] during intestinal transplantation, where the authors noticed that the microbiota of obese mice could reproduce the obese phenotype in a skinny mouse exempt of intestinal microbes. However, the exact nature of the mechanisms and changes involved in the composition of gut microbiota associated with obesity in humans and other animals remains controversial and is not fully understood [13,14]. Within the extensive literature, two main strategies have been used to manipulate the intestinal microbial composition and selectively stimulate the growth and activity of certain species, these being the administration of either prebiotics or food supplements containing living bacteria such as probiotics [15]. Probiotics are individual or multiple living bacterial species, mostly used as feed additives, which can confer a health benefit to the host by improving its microbial balance as well as by modifying the gut microbiota, therefore playing beneficial effects on lipid and glucose metabolism [16]. Probiotics have numerous other functions, including maintaining normal intestinal microorganisms, protecting animals against gastrointestinal disorders, increasing feed efficiency and body weight gain, and improving immune system among others [17,18]. Accordingly, several animal studies have indicated that certain probiotics, including Lactobacilli and Bifidobacteria can suppress body weight gain in rodents [19,20,21], while some strains of probiotics have little effect or promote weight gain [22,23,24]. The anti-obesity potential effect of probiotics seems to depend on the strains used and the underlying mechanisms leading to their effects remain unclear and worthy of investigation. Using animal models, fundamental research has been able to investigate certain of those mechanisms underlying disease processes such as obesity and metabolic syndrome disorder. Indeed, as herbivorous animals, rabbits develop hyperlipidemia rapidly because they are sensitive to high-fat diets [25] and have specific plasma lipid transfer protein (CETP) and low-density lipoprotein (LDL) profiles [26]. In fact, due to the interesting physiological status in terms of lipoprotein metabolism, rabbits are a good and emerging model to investigate obesity and metabolic syndrome. However, this species has not been widely used under high-fat and high-sugar diets to induce obesity or metabolic syndrome disorder [27]. Thus, due to the lack of studies on the impact of probiotics on the diversity of the gut microbiota of obese animals, particularly on ITELV2006 strain rabbits, this study aimed to characterize an experimental model of obesity induced by a diet called “cafeteria” and the different parameters defining metabolic syndrome. It further aimed to evaluate the main consequences on certain biochemical and morphometric parameters of the ITELV2006 strain rabbits by testing the effect of two strains of probiotics, these being *Bifidobacterium animalis subsp. lactis* BB-12^®^ and *Lactobacillus plantarum* 299v^®^.

## 2. Materials and Methods

This trial was organized in two experiments. Both of them were carried out according to the Algerian guidelines for animal care and use. Experiment 1 aimed to first induce obesity and deduce metabolic syndrome on the rabbits that were then characterized for both blood biochemical and morphometric parameters. Experiment 2 used the obese animals from the first experiment to evaluate the effect of two strains of probiotics on the same parameters. Both experiments and all procedures were approved by the Scientific Council of Institute of Veterinary Sciences School of Constantine at Université Frères Mentouri Constantine 1, Algeria under the ethics code UNIV-VS-2019-005 and performed by fully trained personal.

### 2.1. Experiment 1

#### 2.1.1. Animals and Breeding Conditions

A total of 40 rabbits, 20 females and 20 males, from the line ITELV2006 were used. This line was created by cross-breeding rabbits from the local Algerian population (Algerian female) and the INRA 2666 strain as described by Gacem et al. [28]. Rabbits of 855 ± 0.18 g and same age (5 weeks) at the beginning of the experimental protocol were randomly selected. The animals were placed in individual cages (40 cm × 98 cm × 57cm) arranged in a flat deck layout to avoid competition between them in front of food. They were housed in a room with appropriate humidity (50 ± 5%) and temperature (20 ± 1.5 °C), and under a cycle of 12 h controlled lighting. After an acclimatization period of two weeks during which the animals were fed with a standard rabbit diet, they were then randomly divided into equal numbered groups, with 20 rabbits per group, these being the normal control group, “Control”, and the high-fat induced obesity group, “Obese”.

The rabbits of the “Control” group followed an appropriate “ad libitum” feeding, corresponding to rabbits’ energy requirements for maintenance. The rabbits in the “Obese” group were also all fed “ad libitum” for 14 weeks using the “cafeteria” diet. Briefly, this feeding regimen is high in calories and fat, which induces over eating and provides the animals with free access to ‘cafeteria-type foodstuffs’, which consists of ultra-processed and energy dense foods known to provoke obesity. The diet is composed of 50% of the standard diet and 50% of a mixture of food bought in the supermarket, consisting of pâté, biscuits, cheese, crisps, chocolate, peanuts with the respective proportions of 2:2:2:1:1:1 according to the protocol of Darimont et al. [29]. The caloric intake consists of 47% lipids, 27% carbohydrates and 26% proteins. In addition, food intake was recorded daily and assessed with respect to the amount of the ingested feed for each rabbit.

#### 2.1.2. Plasma Measurements

To evaluate the concentrations of important plasma biochemical parameters representative of hepatic, muscular, lipidic, and energetic metabolism, blood samples were taken from all the rabbits from the atrial vein as in [30] prior to diet administration and during weeks 8 and 14. After 7 h of fasting, samples were collected in heparinized tubes, stored on ice and centrifuged (1500 rpm/15 min). The plasma was subsequently extracted and stored at −80 °C until analysis. The fasting blood sugar (GLU), total cholesterol (TC), low-density lipoprotein (LDL), high-density lipoprotein (HDL), triglycerides (TG), creatinine (CREA), total proteins (TP), albumin (ALB), aspartate aminotransferase (ASAT), alanine aminotransferase (ALAT), alkaline phosphatase (AP) were carried out using an autoanalyzer (ADIVA 1800; Siemens, Berlin, Germany) [31].

#### 2.1.3. Morphometric Measurements

Live body measurements were obtained on all rabbits by mean of a tape measure and weighing scale prior to the administration of the experimental regime at day 0 (control) and 8 and 14 weeks. Body weight and abdominal circumference were evaluated weekly following the procedures previously described [30,32]. The Body Mass Index (BMI) for each rabbit was computed using the following formula [33]:

BMI = Body weight (kg) [body length (m) × height (m)]^−1^ (1)

#### 2.1.4. Oral Glucose Tolerance Test (OGTT)

The Oral Glucose Tolerance Test or Oral Induced Hyperglycemia (OGTT) is a test that assesses the ability of body of the animal to lower blood sugar levels after glucose loading. After 16 h fasting [34], a 60% glucose bolus (0.6g/kg bodyweight) was administered by gavage and blood samples were withdrawn from the marginal vein before and at different times after injection (0, 30, 60, 90, 120 and 180 min) as previously described [30]. The OGTT was performed at different times, before administration of the experimental regime at day 0 and during the 8th and 14th weeks with a glucometer Accu-Chek Performa^®^ (Roche Diagnostics, Penzberg, Germany) [30,35].

### 2.2. Experiment 2

This second part of the current trial, as stated above, examined the effect of two strains of probiotics, these being *Bifidobacterium animalis* subsp. *lactis* BB-12 and *Lactobacillus plantarum* 299v on the same biochemical and morphometric parameters described above (experiment 1). Thus, at the end of the experiment 1, 18 rabbits from the “Obese” group were categorized randomly using the standard = RAND() function in Microsoft Excel into three sub-groups of six rabbits (three females and three males) by producing homogenous new populations in terms of weight and Body Mass Index (data not shown). The obese rabbits received probiotics diluted in 1 mL of sterile water by mouth (gavage), every day during a period of one month following the procedure previously described [34]. *Bifidobacterium animalis* subsp. *lactis* BB-12^®^ and *Lactobacillus plantarum* 299v^®^ were purchased from Chr. Hansen Holding A/S (Horsholm, Danemark) and probi AB (Ideon, Lund, Sweden), respectively. The dose used in the present trial was equivalent to the recommended *B. animalis* BB-12^®^ (1 × 10^9^ CFU/mL) and *L. plantarum* 299v^®^ (1 × 10^10^ CFU mL) single daily human dose by each laboratory. For a control test, samples from the water were verified by plating on MRS medium under anaerobic condition, to confirm the viable count of both strains, which contain a final dose of 1 × 10^9^ CFU/mL and 1 × 10^10^ CFU mL, respectively.

A superiority trial approach was used to determine the efficacy of probiotics compared to the control group. Therefore, the three new experimental groups created using the obese rabbits from the experiment 1 were as follow:

(i) Group 1: Obese witnesses (TO) rabbits (*n* = 6) used as control and without any probiotic in their feed.
(ii) Group 2: Obese (OL) rabbits (*n* = 6) given 1 × 10^10^ CFU mL of *Lactobacillus plantarum* 299v^®^ [36].
(iii) Group 3: Obese (OB) rabbits (*n* = 6) receiving 1 × 10^9^ CFU/mL of *Bifidobacterium animalis* subsp. *lactis* BB-12^®^ [35].

All rabbits were fed “ad libitum” with a standard diet during the one month of trial. The morphometric and plasma measurements as well as OGTT were monitored at different times, i.e., before the start of the administration of the probiotics, and 15 days and 30 days after administration, following the methods described above.

### 2.3. Statistical Analyses

All the statistical analyses were performed using XLSTAT 2018.1.1 (AddinSoft, Paris, France). The accuracy of the data, the residues and thus the homoscedasticity of the errors were checked with Levene’s and Breusch-Pagan’s tests, and the normality of the error distribution was appraised using a Kolmogorov–Smirnov test. The analyses were performed using the repeated measures analysis of variance (RMANOVA). The first model tested the fixed effects of group (obese and control), sex (female and male) and time (8 and 14 weeks) and all interactions for the morphometric and plasma biochemical parameters after induction of obesity in rabbits from the Experiment 1. The second model tested the fixed effects of group (TO (obese control), OL (obese rabbits with *L. plantarum* 299v probiotic) and OB (obese rabbits wit *B. animalis* subsp. *lactis* BB-12 probiotic)), sex (female and male) and time (0, 15 and 30 days) and all interactions for the morphometric and plasma biochemical parameters after probiotics supplementation in rabbits from experiment 2. Least squares means were generated for all interactions and main effects analyzed in both experiments. The results following multiple comparisons post-hoc (first with Bonferroni’s test and then confirmed by Tukey test) were considered significant at *p* < 0.05. Values are presented as means ± SEM.

## 3. Results and Discussion

### 3.1. Experiment 1

The analysis of the biochemical and morphometric parameters at day 0, during (at 8 weeks) and after induction of obesity in the rabbits at 14 weeks made it possible to observe significant differences (*p* < 0.001) that are described and discussed in the following sections. In fact, the results of this first experiment showed that the cafeteria diet induced for 14 weeks caused a significant increase in the fasting blood sugar and the OGTT (*p* < 0.001), as well as an alteration of the lipid profile revealed by a significant increase in triglycerides, cholesterol and LDL (*p* < 0.001) and a significant decrease in HDL level (*p* < 0.001). Moreover, no significant difference was observed for ASAT and ALAT. Significant increases in weight, abdominal circumference, length, height, and BMI were also observed. The results of the morphometric parameters revealed that there was a pre-obesity determined by a BMI > 30 (33.29 ± 3.15 Kg/M^2^), an increase in weight (3804 ± 345 g) and an abdominal circumference >40 (47.1 ± 0.96 cm). In addition to these, the significant increase in fasting blood sugar, OGTT, triglycerides, cholesterol and LDL together with the significant decrease in HDL made it possible to confirm the occurrence of the metabolic syndrome.

#### 3.1.1. Fasting Blood Sugar and Oral Glucose Tolerance Test

**Figure 1:**
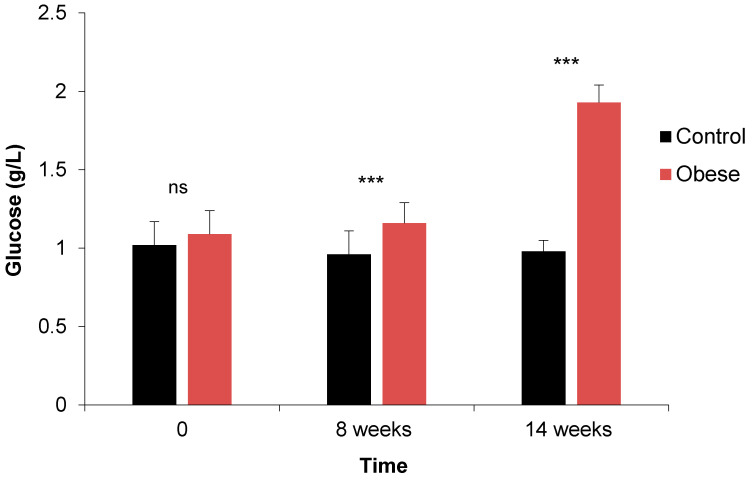
Comparison of fasting blood sugar of rabbits between control and obese groups at day 0 and at 8 and 14 weeks in experiment 1. Significances: *** *p* < 0.001; ns: not significant.

The comparison of the results of blood glucose level in the “obese” group to their control littermates during the period of the trial is given in Figure 1. Furthermore, significant interactions were detected over time after obesity induction, especially within the interaction group × time (*p* < 0.001) (Table 1). However, no significant differences were found within males and females between the two groups.

Regarding OGTT results, normal blood glucose levels increased irrespective of the sex in obese rabbits compared to the controls: 1.17 ± 0.13 g/L in obese vs. 0.96 ± 0.14 g/L for the controls at week 8 and 1.93 ± 0.11 g/L in obese vs. 0.98 ± 0.07 g/L for the controls at week 14 (based on mean values for both males and females). In addition, at the 8th and 14th weeks, obese rabbits had higher blood glucose values and a lower rate of blood glucose clearance compared to control animals, at all the times studied from 30 min to 180 min. OGTT test showed that animals in the control group had blood glucose levels similar to normal values after 180 min (Figure S1). However, this parameter remains high after 180 min in the obese rabbits at the 14th week compared to the controls **(**Figure S1). Significant differences were found within groups and time from 0 to 180 min (*p <* 0.001), and also one interaction for Time × Group, except at T_60_ min (T_0_, T_90_, T_120_, T_180_; *p <* 0.001) and T_30_ (*p <* 0.01), as depicted in Table 2. It is important to note that there was no significant difference within males and females between the two groups.

Fasting Blood Sugar

The results of this parameter indicated a slight increase in fasting blood sugar at the 8th week, followed by hyperglycemia at 14th week in obese rabbits. This result is in accordance with previous studies [30] that noted the occurrence of hyperglycemia when New Zealand rabbits were subjected to a high-fat diet during 28 weeks. However, the values obtained in the present study were different from those reported by Yin et al. [37], who included 37% sucrose in the diet of New Zealand rabbits during a long period of 5 months. Furthermore, according to other earlier studies, there is no significant difference in fasting blood sugar after the administration of high-fat and sucrose diets in the case of Japanese white [38] and the male Watanabe heritable hyperlipidemic type rabbits [39].

OGTT

The defects in blood glucose regulation were observed as depicted by the OGTT results. Indeed, the increase in fasting blood sugar after 180 min for the “obese” group reflects the inability of the rabbit body to recover normal glucose levels. The precise mechanisms of action underlying these anomalies are not completely understood [38]. For instance, previous studies have shown that visceral adipose tissue in obese animals and humans can cause an increased inflow of fatty acid throughout the portal vein into the liver as a result of a high-fat diet, which may originate in an insulin resistance state [40,41]. In addition, insulin resistance is characterized by a failure of cells and tissues to respond appropriately to insulin in the presence of a normal concentration of insulin or as a normal response to hyperinsulinism [42]. Other studies have reported that the adipocytes of obese rabbits are larger than those of normal rabbits [43], which reduces their ability to absorb and oxidize glucose in comparison to small adipocytes in presence of insulin [44]. These findings may explain the hyperglycemia observed in the obese rabbits of the present study. An earlier study by Saisho [45] reported that a period of around 100 days under a “cafeteria” diet is capable of developing a state of pre-diabetes followed by alteration in both blood sugar level during fasting and glucose intolerance. Certainly, even though rabbits did not develop type 2 diabetes, the model could be useful to study the condition that precedes the clinical manifestation of the pathology, allowing the identification of preclinical markers, required for the detection of patients at risk.

#### 3.1.2. Lipids

A significant increase (*p* < 0.001) was recorded from the 8th and the 14th week in the “obese” group compared to the controls for total cholesterol (TC) (Figure S2A), LDL (Figure S2B) and triglycerides (TG) (Figure S2C). Inversely, a significant decrease (*p* < 0.001) in HDL was observed for the obese rabbits (Figure S2D). Furthermore, significant interactions for LDL, HDL, total cholesterol and triglycerides were observed over time for the “obese” group from the 8th until the 14th week (Time × Group, *p* < 0.001) (Table 1). As for the results above concerning glucose and OGTT, no significant difference (*p* > 0.05) was found between males and females of the two groups.

The obesity caused by the “cafeteria” diet led to dyslipidemia. These results are in agreement with those described in similar studies to our trial [30,46], which observed that both triglycerides and cholesterol levels are higher in obese rats and rabbits compared to controls littermates that were subjected to a standard diet. Similar effects on blood cholesterol level of rabbits fed high-fat and cafeteria diets were also observed in other studies [47,48], Thus, the hyperlipidemia observed when rabbits were fed the “cafeteria” diet can be explained by both the high fat content of the diet and its caloric content released from fat [27]. Among all macromolecules, fat is an ester generally known as a triglyceride composed of three chains of fatty acids and glycerol. Large amounts of glycerol and fatty acids are freely mobilized in the bloodstream. Fatty acids are also the main substrates for the production of Very Low-Density Lipoprotein (VLDL) and Low-Density Lipoprotein (LDL) in the liver [49]. These changes in the composition of lipoproteins are well known to be related to an increase in the activity of hepatic 3-hydroxy-3-methylglutaryl coenzyme A reductase, an enzyme involved in the synthesis of cholesterol. Thus, most free fatty acids are synthesized in the liver and adipose tissue to form new triglycerides or new fat reserve, a metabolic event known as lipogenesis [50].

#### 3.1.3. Total Protein, Creatinine and Albumin

A significant increase in the total protein level was observed for the “obese” group (*p* < 0.01) at the 14th week (*p* < 0.001) (Table 1). Creatinine increased also significantly (*p* < 0.001) at both the 8th and the 14th week (Table 1). Furthermore, a significant difference in albumin levels was found over time within all the animals (*p <* 0.01) (Table 1). It is important to note that no significant difference was detected within males and females between the obese and control rabbits.

The increase in total proteins in obese rabbits from the 8th to the14th week of this trial was not due to an increase in albumin for which we observed constant concentrations in both rabbit groups. We might partly attribute this increase to an increase in globulins, which have recently been found to be linked to the development of type 2 diabetes [51]. Therefore, they have been suggested as biomarkers for the development of hepatic fibrosis in patients with non-alcoholic fatty liver disease [52]. The results observed in this trial are similar to the results reported in previous experiments [30]. For example, in this latter study, the authors induced obesity in New Zealand rabbits using a diet with high in fat and sucrose.

The increase in the level of albumin and creatinine from the 8th until the 14th week, for all the rabbits, may be on the other hand a consequence of age effect. Accordingly, animals displayed low values compared to aged animals during the weeks following the experiment [53,54]. Some trials suggested that a significant relationship might exist between weight and creatinemia ratio [55].

#### 3.1.4. Aspartate Aminotransferase (ASAT) and Alanine Aminotransferase (ALAT)

In the present study, no significant differences (*p* > 0.05) in ASAT, ALAT, and the ASAT/ALAT ratio were found between the two groups over time (Table 1). However, a significant difference was observed for sex × group interaction for ASAT and ASAT/ALAT ratio (*p <* 0.001).

According to Zhao et al. [38], the activity levels of ALAT, ASAT and the serum ASAT/ALAT ratio are those commonly used specifically to identify liver damage in domestic animals and to detect biliary obstruction (mild and progressive liver damage). Thus, the non-modification of these blood parameters between the two groups during the 14 weeks of the experiment can be for instance explained by the absence of liver damage. However, it is worthy to note that the findings of a study similar to the experimental design of our trial [30] showed a significant increase in ASAT and ASAT/ALAT ratio, but after a longer period of 28 weeks of induction using a diet rich in fat and sucrose [30]. Based on previous statements, the results observed in our trial may be related to the extent of the administration of the diet [56].

#### 3.1.5. Alkaline Phosphatase

For this important biochemical parameter, a significant decrease in alkaline phosphatase was observed at the 8th and 14th weeks for all the rabbits (*p <* 0.001) (Table 1), but without any effect of sex, whatever the group. Alkaline phosphatase is known to have high activity in cartilage undergoing endochondral ossification [57,58]. This enzyme plays also a pivotal role in the process of hard tissue mineralization [59,60], therefore explaining the higher levels observed in young rabbits currently being in growth at the beginning of the experiment (7 weeks), compared to adults at the end of the first experiment (4 and a half months). Our findings are further in agreement with previous experiments that reported no sex effect on alkaline phosphatase [55].

#### 3.1.6. Morphometric Parameters

**Figure 2:**
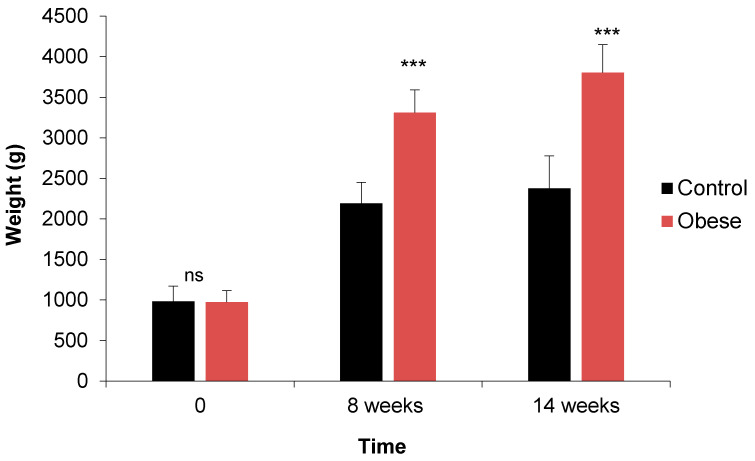
Comparison and evolution of weight of the rabbits between control and obese groups at D0, 8 and 14 weeks in experiment 1. Significances: *** *p* < 0.001; ns: not significant.

A significant increase (*p <* 0.001) was observed in the “obese” group for abdominal circumference, BMI, weight and abdominal circumference/length ratio (Table 3). However, no significant difference (*p* > 0.05) was noted for length and height. At the 8th week, significant differences were observed for all the morphometric parameters evaluated in this study (Table 3). Inversely to the biochemical parameters, significant differences were detected for females for BMI (*p <* 0.01) and weight (*p* < 0.001) (Table 3). Interactions of time × group were found for abdominal circumference (*p* < 0.001), abdominal circumference/length ratio (*p* < 0.01) and of time × sex for weight (*p <* 0.001) (Table 3). The weekly monitoring of body weight for 14 weeks, in the two groups of rabbits allowed us to evaluate in a precise manner the weight gain during this induction period. We can observe, as expected, a progressive and significant weight gain within both groups (control and obese). However, the rabbits of the “obese” group and whatever the sex showed a greater increase in body weight compared to their littermates (3310 ± 280 vs. 2191 ± 258 g, *p* < 0.001) at the 8th week. At the end of the 14th weeks of the trial, this increase stills significant and reached 3804 ± 345 g for the obese compared to the control (*p* < 0.001) (Figure 2).

The induction of obesity in the present study was characterized by an increase in body weight in rabbits fed the “cafeteria” diet, compared to control rabbits fed a standard diet. Indeed, a respective increase of 58 and 61% of the body weight and the BMI was observed at week 14, showing that the rabbits were in moderate obesity and far from a severe degree of obesity. Similar findings were observed by Carroll et al. [61] with around 45% increase in body weight in young male rabbits fed a high-fat diet, while Arias-Mutis and co-workers [30] found a weaker increase of 24% and 22% of body weight and BMI, respectively, in adult New Zealand rabbits. Further data indicated that an induced high-fat diet for almost two months might cause an increase in food intake, body weight and accumulation of lipids in adipose tissue [62]. In contrast, Brunner et al. [63] did not observe any significant difference in weight of the rabbits that were fed a diet rich in fat.

Our study also revealed a significant increase in the abdominal circumference and the abdominal circumference/length ratio. According to Hariri et al. [64], the fatty acid composition of foods can play a role in the regulation of body weight. Studies in both animals and humans have shown that polyunsaturated fatty acids are more easily used as fuel, while saturated fatty acids are more likely to be accumulated in fatty tissue. Therefore, the gain in body weight may be in part explained by the increase in abdominal fat mass [30]. Similarly, the significant differences detected in females for BMI and weight can be related to a predominance of obesity in females compared to males. Indeed, estrogen in females facilitates the storage of fat [65].

#### 3.1.7. Overall Discussion of the Impact of Cafeteria Diet

The results of the first experiment (induction trial) showed that the high-fat and high-calorie “cafeteria” diet administered for 14 weeks was well tolerated by the rabbits. In fact, the weight of the rabbits gradually increased until the end of the induction protocol, which made it possible to detect central obesity, a state of pre-diabetes characterized by impaired fasting blood sugar, a worsening of the glucose intolerance and an alteration of the lipid profile revealed for instance by an increase in triglycerides, cholesterol and LDL and a decrease in HDL.

Many researchers have used different high-fat diets with levels varying between 10 and 60% to induce obesity and MetS in experimental models and their ability to induce obesity has been demonstrated by numerous studies [26,66,67]. For example, Zarzoso et al. [32] fed the rabbits with a high-fat diet for 18 weeks and revealed that the body weight and blood sugar level were higher in the high-fat diet group compared to the control rabbits. Other studies in rats have shown that a 64% high sugar (sucrose) level in the diet does not lead to a difference in weight even until a period of 80 days after the beginning of the diet [68]. Another study by Romestaing et al. [69] showed that the use of diets enriched with coconut oil or butter does not induce obesity but induce an increase in adipose mass after 14 weeks. In addition to the above, another earlier study comparing the effect of 45% High-Fat Lard diet versus to “cafeteria” diet in male rats revealed, in agreement with our findings, an increase of body mass in animals fed with the cafeteria diet. Indeed, after 7 weeks of diet, the animals of the “cafeteria” group presented hyperinsulinemia, glucose intolerance and insulin resistance compared to the animals of the High-Fat Lard group [70]. It is worthy to note that these authors observed marked obesity in the animals of the “cafeteria” group compared to animals fed the Lard diet. Overall, our results, and in comparison to the extensive literature, demonstrate that the “cafeteria” diet used in this trial is an ideal model for causing obesity.

#### 3.1.8. Overall Discussion Concerning the Induction Obesity and Metabolic Syndrome

In this first experiment, the use of the “cafeteria” diet for 14 weeks induced, according to Grundy [8], moderate obesity, and consequently a metabolic syndrome, as evidenced by four parameters of five tested, including (i) abdominal obesity, (ii) dyslipidemia with a decrease in HDL, (iii) an increase in total cholesterol, triglycerides and LDL, and (iv) glucose intolerance and pre-diabetic condition. The results obtained in our study are similar to the criteria that have been established in humans for the diagnosis of metabolic syndrome [71]. Hepatic steatosis is not included in the MetS diagnostic criteria, but often follows the results of the metabolic abnormalities. Other results [72] demonstrated that MetS was developed upon hyper-triglyceridemia, a decrease of HDL and glucose intolerance in male rats fed the cafeteria diet during a period of 3 months.

### 3.2. Experiment 2

The results showed an improvement from day 15 of the administration of probiotics for the two groups (OB and OL) compared to the control group, on the parameters of obesity and metabolic syndrome induced during the first experiments. Thus, a significant decrease in fasting blood sugar (*p* < 0.001), OGTT (*p* < 0.05), total cholesterol (*p* < 0.001), triglycerides (*p* < 0.01) and LDL (*p* < 0.001), a significant increase in HDL (*p* < 0.05) and a significant decrease in weight (*p* < 0.05) (3523 ± 521 g for the OL group and 3498 ± 117 g for OB), abdominal circumference (*p* < 0.05) (46 ± 1.36 cm for OL and 45 ± 1.33 cm for OB), and BMI (*p* < 0.05), by increasing the time of exposition to the probiotics. In contrast, no significant difference was observed between the two probiotics. The biochemical markers and morphometric parameters evaluated for the three groups of rabbits, after 4 weeks of administration of the probiotics are reported in Table 4 and Table 6.

#### 3.2.1. Fasting Blood Sugar and Oral Glucose Tolerance Test

**Figure 3:**
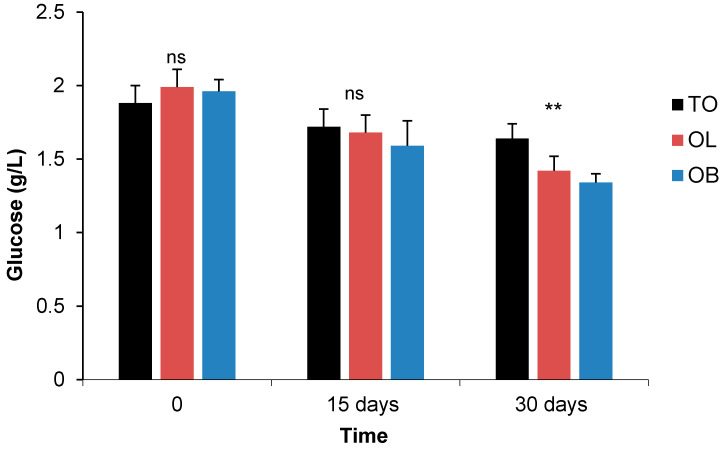
Comparison of fasting blood sugar of rabbits between control obese rabbits and treated groups with probiotics at D0, 15 and 30 days in experiment 2. Significances: ** *p* < 0.01; ns: not significant. Groups: TO: Control Obese; OB: Obese + *Bifidobacterium animalis* subsp. *lactis* BB-12 probiotic; OL: Obese + *Lactobacillus plantarum* 299v probiotic.

Our findings revealed that blood glucose level decreased significantly (*p <* 0.05) in the OL and OB groups compared to the control group (Table 4). Moreover, a greater decrease was observed for the OB group for which a *B. animalis* subsp. *lactis* BB-12 probiotic was administered. This was found to be highly significant from the 15th until the 30th day after the administration of the probiotics (*p* < 0.001), particularly for the OB group, irrespective of the sex, at day 30 (*p* < 0.01) compared to the control group (1.7 ± 0.12 control vs. 1.68 ± 0.12 (OL) vs. 1.59 ± 0.17 g/L (OB) from the 15th day and (1.64 ± 0.10 control vs. 1.42 ± 0.10 (OL) vs. 1.34 ± 0.06 g/L (OB)) on the 30th day, as clearly depicted in Figure 3. For this parameter, a significant time × group (*p <* 0.01) interaction was found (Table 4), but no significant difference was observed between the females and male whatever the group.

The OGT test was performed during and after the administration of the probiotics for 4 weeks. On day 0, the fasting glucose level of all the obese rabbits was high, then a small decrease in glycemia was observed for the OB and the OL groups compared to the obese control animals from the 15 days (Figure S3A). The OB rabbits showed lower blood glucose values than the OL rabbits at all-time points studied after 30 min of glucose, and this from the 15th until 30th day (Figure S3). At 180 min, the OB and OL groups showed blood sugar levels below the baseline compared to the control group after 15 days (Figure S3F). Further significant differences (Table 5) were observed within the group for OGTT at 0 min (*p <* 0.001) for the OB and OL compared to the control and OGTT at 120 min (*p <* 0.05) for the OB group compared to the control and OL groups. For this parameter, a sex effect was identified at T0 (*p* < 0.001), T30, and T180 min (*p <* 0.01). The sex × group interaction for OGTT was only significant at 0 min (*p <* 0.01), 60 min (*p <* 0.01) and 120 min (*p <* 0.001) (Table 5).

Fasting Blood Sugar

The level of fasting sugar in the blood decreased significantly in the OL and OB groups compared to the control group. These results are consistent with those obtained by Zhang et al. [35] in diabetic animal models during the administration of *L. plantarum strain* 299v (5 × 10^7^ CFU/mL). These results are also similar to those of other studies, and for example to those of Tonucci et al. [73] who noted a significant decrease in fasting blood sugar in 50 individuals following the administration of *L. Acidophilus* La-5 (10^9^ CFU) and *B. Animalis* subsp *lactis* BB-12 (10^9^ CFU/mL) in fermented milk for up to 6 weeks.

OGTT

The OGTT results allowed us to see that the fasting blood sugar levels are lower after the administration of probiotics in the OB and OL groups at 120 min compared to the control, but they remain non-significant. Glucose elimination curves (Figure S3) were similar for the two groups, indicating reduced but non-significant glucose intolerance for the OB group more than the OL group at all the times from 0 to 180 min. These results are in agreement with those reported in some studies such as Sato et al. [74] who found a non-significant reduction in OGTT in rats that consumed *L. gasseri* SBT 2055 at 10^7^ CFU/mL for 4 weeks. Similarly, the impact was not very clear using the same strain, from the study by Hamad et al. [75]. The administration of four *Bifidobacteria* strains named L66-5, B. L75-4, B. M13-4 and B. FS31-12, respectively, at 10^8^ CFU/mL for 6 weeks in male rats made it possible to obtain similar conclusions [76]. Other studies have shown that a 6 week treatment with *B. animalis* ssp. *lactis* 420 at a level of 10^9^ CFU significantly improved glucose tolerance in mice fed a high-fat diet [77]. According to these authors, a supplementation of *Lactobacillus* to a high-fat diet improved glucose tolerance in mice only after 18 weeks of use. However, no significant difference was observed after 4 weeks of this same study. The mechanisms underlying this hypoglycemia effect remain unclear. According to Tremaroli and Bäckhed [11], butyrate is produced by the microbiota during the fermentation process of non-digestible polysaccharides. This molecule functions as an important source of energy for intestinal epithelial cells [78]. Butyrate also has an impact on metabolism, as it has a suppressive effect on the oxidation of glucose in the intestinal cells of rats [79]. This implies that butyrate influences the amount of glucose that is metabolized into energy and the amount converted into fat. It can further affects metabolism by increasing energy expenditure, improving insulin sensitivity and anti-inflammatory properties [80]. An interesting study [81] supports these statements, finding that obese individuals had a reduced concentration of butyrate-producing bacteria compared to lean individuals.

#### 3.2.2. Lipids

The treatment with probiotics allowed us to see that there is a significant decrease of total cholesterol (*p* < 0.001), triglycerides (*p* < 0.01) and LDL (*p* < 0.001) and a significant increase in HDL (*p* < 0.05) from the 15th day upon the administration of probiotics (Table 4). However, no significant difference was observed between the OL and OB groups and between males and females (Table 4). According to an earlier study [36], a significant reduction in total cholesterol was observed after the use of the strain *L. plantarum* 299v at 5 × 10^7^ CFU/mL during 6 weeks. However, insignificant values were recorded by others [77] for triglycerides and HDL levels between groups that received *B. animalis* ssp. *lactis* 420 with a dose of 10^9^ CFU for 6 weeks. In rats, a weak effect was observed on triglycerides between the control group and those that consumed *L. gasseri* SBT2055 at 10^7^ CFU/mL for 4 weeks [75]. Overall, several studies have evidenced that probiotics have hypercholesterolemia effects in rats and humans, such as *B. Bacteriumlongum* BL1 [82] and *L. plantarum* MA2 [83]. Meanwhile, a reduction in total cholesterol and triglyceride levels and an increase in the high density lipoprotein/low density lipoprotein (HDL/LDL) ratio was observed in male rats following administration of several *Bifidobacteria* strains at 10^8^ UFC/mL for 6 weeks [76]. Thus, one can suggest that the mechanisms involved in improving the lipid profile, in particular cholesterol and its fractions may be related only to some probiotics strains, and further studies are needed to better understand the underlying pathways of this dependency association. It is also important to mention that certain strains of probiotics are able to incorporate cholesterol into bacterial cells, to hydrolyze bile salts or to inhibit hydroxylmethylglutaryl-CoA, the enzyme limiting the rate of cholesterogenes, thereby reducing plasma cholesterol [84].

#### 3.2.3. Creatinine, ALB, PT, ASAT, ALAT and AP in Experiment 2

Our findings revealed that there is no significant difference (*p*> 0.05) between the three groups for CREA, ALB, PT, ASAT, ALAT, ASAT/ALAT ratio and AP. However, we observed a significant decrease in males for total protein and ALAT (*p <* 0.05). In addition, a significant and weak sex × group interaction was detected for creatinine (*p* < 0.05) (Table 4). Our findings are overall in accordance with those of earlier studies [85,86,87], who reported that the levels of total serum proteins, albumin, globulin and AP, of rabbits given probiotics were not affected. In a similar experimental design to our trial, a study [88] reported non-significant results in the levels of creatinine in New Zealand rabbits that consumed multiple doses of anaerobic probiotics during a period of 56 days. On the other hand, the rabbits of our trial did not exhibit liver damage, because none of the blood metabolites were altered among the groups that received the probiotics. Importantly, our findings agree with the no effect observed for ASAT and ALAT, as well as the ratio of ASAT/ALAT values, in rats consuming *L. gasseri* SBT2055 at 10^7^ CFU/mL for up to 4 weeks [75]. The significant but weak (*p* > 0.05) difference observed between the males and the females for total proteins is in agreement with the study of Elamin [55], which reported that the concentration in rabbits was less significant in males over 5 months compared to females of the same age.

#### 3.2.4. Morphometric Parameters

**Figure 4:**
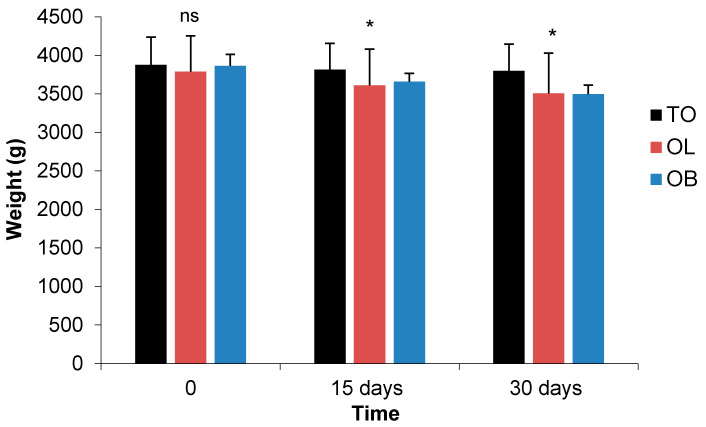
Comparison and evolution of weight of the rabbits between control obese rabbits and the groups treated with probiotics at days 0, 15 and 30 in experiment 2. Significances: * *p* < 0.05; ns: not significant. Groups: TO: Control Obese; OB: Obese + *Bifidobacterium animalis* subsp. *lactis* BB-12 probiotic; OL: Obese + *Lactobacillus plantarum* 299v probiotic.

In this second experiment, and from day 15 to 30 after the administration of probiotics, abdominal circumference, BMI and body weight induced by the cafeteria diet from the experiment 1 were significantly reduced by time of exposition (*p* < 0.05). In fact, the groups of rabbits that were fed with *Lactobacillus plantarum* 299v and *Bifidobacterium animalis* subsp. *lactis* BB-12 probiotic strains showed a decrease in body weight compared to the control group (Figure 4).

A sex × group interaction was observed for length (*p* < 0.001), abdominal circumference/length ratio (*p* < 0.01), BMI and weight (*p* < 0.05). The significant decrease of length, BMI and weight was observed for males (Table 6). Generally, our results are in agreement with earlier studies [77] that evidenced a significantly reduced body weight in mice fed with a high-fat diet after treatment by *B. animalis* ssp. *Lactis* 420 at 10^9^ CFU during 6 weeks. A combination of two probiotics, these being *L. acidophilus* and *B. bifidus*, was also found to be able to reduce the weight [89]. However, the use of *L. plantarum* 299v strain at a dose of 1× 10^9^ CFU/mL over 6 weeks on 30 men of good health was not able to induce any weight loss [36]. Equivalent results using the same strain with an amount of 1 × 10^8^ CFU/mL for 5 weeks in rats fed high cholesterol diets have been reported [83].

The significant decrease of BMI and weight observed in males, especially for the OL group, can be explained by the body fat content, which is 20 to 25% in females compared to 10–15% in males, whatever the weight, whereas the lean muscle mass in males was more important. Therefore, we confirm that the body must burn more calories to maintain the overall muscle mass metabolism and weight [65]. Moreover, the weight decrease can be explained by the administration of probiotics, which lead to the reduction in the proportion of Gram-negative microorganisms in the microbiota. This can affect the decrease in the level of lipopolysaccharides in the bloodstream that in turn reduces the storage of lipids in adipose tissue, which all together induce a reduction in the weight gain [90]. It is worthy to mention that different clinical studies are difficult to compare due to differences in study sizes, study populations, strains, dosages of probiotics used and how they were administered. In this trial, the short duration of the study (4 weeks) could also be the reason for the weak of effects on the final values of abdominal circumference, BMI and weight. On the other hand, according to other authors, the future treatment of obesity with probiotics should contain a cocktail of several microorganisms [91].

#### 3.2.5. Overall Discussion about the Effect of Probiotics Supplementation on Obesity and Metabolic Syndrome

In the present study, treatment with two probiotics, these being *B. animalis* subsp. *Lactis* BB-12 and *L. Plantarum* 299va, demonstrated an improvement in certain parameters of induced obesity and MetS perceived during the first experiment. In fact, the anti-obesity potential of probiotics has been further confirmed. There was a significant decrease *(p* < 0.05) in weight, BMI and abdominal circumference with time for the groups that received probiotics compared to the controls. Studies on animals have shown that the consumption of probiotics could treat and/or prevent obesity [90]. In this latter study, in which the authors focused on clinical trials to examine the effect of specific microorganisms on body weight control, the results indicated that *L. gasseri* SBT2055, *L. rhamnosus* ATCC53103 and the combination of *L. rhamnosus* ATCC 53,102 and *B. lactis* Bb12 may reduce obesity, body weight and weight gain [90]. Furthermore, the probiotics used in our study improved the four parameters of MetS detected during the first experiment. Another study that used the *B. Pseudocatenulatum* CECT 7765 strain in obese insulin-resistant mice reduced metabolic syndrome through a reduction in weight gain and glucose intolerance [91]. Furthermore, the administration of *B. lactis* 420 reduced MetS by decreasing weight and glucose intolerance in obese and diabetic mice [77]. Overall, recent studies suggest that manipulating the composition of the microbial ecosystem in the gut may be a novel approach in the treatment of obesity [92]. Thus, such a treatment could consist of modifying the composition of the microbial community of an obese individual by the administration of beneficial microorganisms. The contradictory results concerning the effects of probiotics on body weight may be due to the animal model used (rabbit, mouse, rat), the duration of treatment, the number of colonies used and the protocol for administering of the probiotics. Further studies are also needed to better understand the underlying mechanisms.

## 4. Conclusions

A relevant model of obesity induced by giving a “cafeteria” diet for 14 weeks demonstrated a change in biochemical and morphometric parameters, reproducing a model of pre-obesity and the main clinical manifestations of the metabolic syndrome. Then, the treatment with two probiotics, these being *B. animalis* subsp. *lactis* BB-12^®^ and *L. Plantarum* 299va^®^, demonstrated an improvement in certain induced parameters of obesity and MetS determined during the first experiment. This study revealed that the used probiotics could exert beneficial effects, but in a dependent manner, for the rabbit strain we used in this trial. The anti-obesity potential is established by this preliminary study, as significant results were obtained against BMI, weight and abdominal circumference. Moreover, the metabolic syndrome in obese rabbits fed with the high-fat and high-calorie “cafeteria” diet was demonstrated by inducing a significant improvement in some MetS parameters such as fasting sugar, OGTT, total cholesterol and LDL, HDL and triglycerides. In this trial, no significant difference was observed between the effects of the two probiotics. Although many studies have recognized that probiotics could be useful in the treatment of obesity and metabolic syndrome, questions remain unanswered regarding the specific strains and the dosage to be administered. Thus, further research should be directed towards a combination of two or more probiotics with different strength and/or duration of administration, in order to reduce and to treat the obesity and the metabolic syndrome in rabbits as well as in other species.

## Figures and Tables

**Table 1:** Results of the comparisons of the biochemical parameters for the effects of rabbit group (G), sex (S), time (T) and the interactions from experiment 1.

Parameters ^1^	Sex	Control			Obese			Effects ^2^						
		0	8	14	0	8	14	Group ^3^	Time	Sex	S × T	G × T	S × G	G × T × S
GLU g/L	M	1.02 ^bc^	0.93 ^c^	0.99 ^bc^	1.12 ^bc^	1.18 ^b^	1.91 ^a^	***	***	ns	ns	***	ns	ns
	F	1.02 ^bc^	0.99 ^bc^	0.99 ^bc^	1.08 ^bc^	1.16 ^b^	1.96 ^a^							
TC (g/L)	M	0.66 ^def^	1.14 ^de^	1.23 ^d^	0.46 ^f^	1.86 ^bc^	2.62 ^a^	***	***	ns	ns	***	ns	ns
	F	0.55 ^f^	1.17 ^de^	1.23 ^d^	0.61 ^ef^	1.84 ^c^	2.44 ^ab^							
HDL (g/L)	M	0.16 ^cde^	0.24 ^abc^	0.29 ^ab^	0.14 ^de^	0.19 ^cde^	0.17 ^cde^	***	***	ns	ns	***	ns	ns
	F	0.15 ^cde^	0.25 ^abc^	0.32 ^a^	0.14 ^e^	0.19 ^cde^	0.17 ^cde^							
LDL (g/L)	M	0.65 ^bc^	0.78 ^bc^	0.95 ^bc^	0.64 ^bc^	0.85 ^bc^	1.46 ^a^	***	***	ns	ns	***	ns	ns
	F	0.58 ^c^	0.86 ^bc^	0.86 ^bc^	0.65 ^bc^	1.01 ^b^	1.48 ^a^							
TG (g/L)	M	0.34 ^c^	0.43 ^c^	0.49 ^c^	0.28 ^c^	1.23 ^b^	3.30 ^a^	***	***	ns	ns	***	ns	ns
	F	0.29 ^c^	0.41 ^c^	0.49 ^c^	0.36 ^c^	1.21 ^b^	3.30 ^a^							
CREA (mg/L)	M	4.87 ^b^	12.19 ^a^	12.57 ^a^	5.01 ^b^	11.98 ^a^	12.85 ^a^	ns	***	ns	ns	ns	ns	ns
	F	5.56 ^b^	12.46 ^a^	12.76 ^a^	5.06 ^b^	12.82 ^a^	13.15 ^a^							
TP(g/L)	M	56.15 ^abc^	56.92 ^abc^	58.31 ^abc^	55.04 ^bc^	68.20 ^abc^	79.71 ^a^	**	***	ns	ns	*	ns	ns
	F	48.25 ^c^	52.06 ^c^	59.01 ^abc^	49.59 ^c^	55.28 ^bc^	76.74 ^ab^							
ALB (g/L)	M	27.40 ^b^	40.40 ^ab^	40.76 ^ab^	35.58 ^ab^	45.56 ^a^	45.87 ^a^	ns	**	ns	ns	ns	*	ns
	F	37.83 ^ab^	42.92 ^ab^	43.20 ^ab^	32.29 ^ab^	37.95 ^ab^	38.27 ^ab^							
ASAT(UI/L)	M	43.85	44.89	47.36	52.80	55.34	62.33	ns	ns	ns	ns	ns	***	ns
	F	51.80	52.36	51.18	41.56	44.28	48.28							
ALAT(UI/L)	M	47.58	47.92	47.27	46.39	47.75	47.66	ns	ns	ns	ns	ns	ns	ns
	F	45.74	45.89	45.41	46.11	48.15	47.89							
ASAT/ALAT	M	0.94	0.96	1.03	1.13	1.15	1.29	ns	ns	ns	ns	ns	***	ns
	F	1.19	1.20	1.18	0.92	0.92	1.03							
AP (UI/L)	M	203.00 ^a^	93.40 ^c^	91.20 ^c^	189.50 ^ab^	92.90 ^c^	105.40 ^bc^	ns	***	ns	ns	ns	ns	ns
	F	166.70 ^abc^	92.00 ^c^	100.00 ^bc^	176.90 ^abc^	98.40 ^bc^	106.20 ^bc^							

^1^ Abbreviations: GLU: Fasting blood sugar, TC: Total Cholesterol, HDL: High-Density Lipoprotein, LDL: Low-Density Lipoprotein, TG Triglycerides, CREA: Creatinine, TP: Total Protein, ALB: Albumin, ALAT: Alanine Aminotransferase, ASAT: Aspartate Aminotransferase, AP: Alkaline Phosphatase. ^2^ Significances: ns: not significant; *: *p* < 0.05; **: p < 0.01; ***: *p* < 0.001. The parameters in each row (for males and females) not followed with the same superscript letters are significantly different (*p* < 0.05). ^3^ Control rabbits versus obese rabbits (with hyper caloric and hyper lipidic rich diet).

**Table 2:** Variations of Oral Glucose Tolerance Test (OGTT) evaluated over time on the rabbits in experiment 1 and effects of group (G), sex (S), time (T) and the interactions.

Parameters ^1^	Sex	Control			Obese			Effects ^2^						
		0	8	14	0	8	14	Group ^3^	Time	Sex	S × T	G × T	S × G	G × T × S
OGTT (T0) (g/L)	M	1.02 ^bc^	0.93 ^c^	0.99 ^bc^	1.12 ^bc^	1.18 ^b^	1.91 ^a^	***	***	ns	ns	***	ns	ns
	F	1.02 ^bc^	0.99 ^bc^	0.99 ^bc^	1.08 ^bc^	1.16 ^b^	1.96 ^a^							
OGTT (T30) (g/L)	M	2.32 ^cd^	2.13 ^d^	3.04 ^bc^	2.69 ^cd^	3.55 ^ab^	3.92 ^a^	***	***	ns	ns	**	ns	**
	F	2.16 ^d^	2.52 ^cd^	2.31 ^cd^	2.59 ^cd^	3.01 ^bc^	3.89 ^a^							
OGTT (T60) (g/L)	M	1.33 ^bc^	2.09 ^ab c^	2.31 ^ab c^	2.10 ^ab c^	2.58 ^ab^	3.03 ^a^	***	***	ns	ns	ns	ns	ns
	F	1.32 ^bc^	1.99 ^ab c^	1.95 ^ab c^	1.21 ^c^	2.30 ^ab c^	2.96 ^a^							
OGTT (T90) (g/L)	M	1.11 ^c^	1.50 ^bc^	1.48 ^c^	1.16 ^c^	2.13 ^ab^	2.39 ^a^	***	***	ns	ns	***	ns	ns
	F	1.29 ^c^	1.49 ^c^	1.61 ^bc^	1.11 ^c^	1.74 ^bc^	2.47 ^a^							
OGTT (T120) (g/L)	M	0.97 ^b^	1.10 ^b^	1.17 ^b^	1.01 ^b^	1.11 ^b^	1.83 ^a^	***	***	ns	ns	***	ns	ns
	F	1.00 ^b^	1.10 ^b^	1.01 ^b^	0.92 ^b^	1.11 ^b^	2.02 ^a^							
OGTT (T180) (g/L)	M	0.94 ^b^	0.97 ^b^	0.94 ^b^	1.00 ^b^	0.97 ^b^	1.30 ^a^	**	***	ns	ns	***	ns	ns
	F	0.98 ^b^	0.97 ^b^	1.01 ^ab^	0.94 ^b^	0.95 ^b^	1.30 ^a^							

^1^ OGTT = Oral Glucose Tolerance Test evaluated at 0, 30, 60, 90, 120 and 180 min. ^2^ Significances: ns: not significant; **: *p <* 0.01; ***: *p* < 0.001. The parameters in each row (for males and females) not followed with the same superscript letters are significantly different (*p* < 0.05). ^3^ Control rabbits versus obese rabbits (with hyper caloric and hyper lipidic rich diet).

**Table 3:** Results of the comparisons of the morphometric parameters for the effects of rabbit group (G), sex (S), time (T) and interactions from experiment 1.

Parameters	Sex	Control			Obese			Effects ^1^						
		0	8	14	0	8	14	Group ^2^	Time	Sex	S × T	G × T	S × G	G × T × S
Length (cm)	M	38.40 ^b^	43.90 ^a^	44.50 ^a^	38.90 ^b^	43.50 ^a^	44.60 ^a^	ns	***	ns	ns	ns	ns	ns
	F	39.40 ^b^	43.50 ^a^	44.50 ^a^	38.40 ^b^	43.70 ^a^	45.40 ^a^							
Height (cm)	M	20.00 ^d^	23.50 ^c^	26.00 ^a^	19.70 ^d^	23.40 ^c^	25.20 ^ab^	ns	***	ns	ns	ns	ns	ns
	F	19.90 ^d^	24.00 ^bc^	25.40 ^ab^	19.90 ^d^	23.10 ^c^	25.70 ^a^							
Abdominal Circumference (cm)	M	30.80 ^d^	41.80 ^bc^	42.10 ^bc^	30.80 ^d^	43.30 ^b^	46.90 ^a^	***	***	ns	ns	***	ns	ns
	F	31.20 ^d^	40.60 ^c^	43.10 ^b^	31.20 ^d^	42.80 ^bc^	47.30 ^a^							
Abdominal Circumference/Length ratio	M	0.80 ^c^	0.95 ^b^	0.95 ^b^	0.79 ^c^	1.00 ^ab^	1.05 ^a^	***	***	ns	ns	**	ns	ns
	F	0.79 ^c^	0.93 ^b^	0.97 ^b^	0.81 ^c^	0.98 ^ab^	1.05 ^a^							
BMI ^3^ (kg/m^2^)	M	1.99 ^e^	20.09 ^bc^	18.49 ^c^	12.34 ^d^	32.63 ^a^	32.68 ^a^	***	***	**	ns	ns	ns	ns
	F	2.15 ^e^	22.21 ^bc^	23.09 ^b^	13.26 ^d^	32.89 ^a^	33.92 ^a^							
Weight (g)	M	996 ^e^	2064 ^d^	2138 ^d^	944 ^e^	3308 ^b^	3665 ^ab^	***	***	***	***	***	ns	ns
	F	969 ^e^	2318 ^cd^	2613 ^c^	1004 ^e^	3311 ^b^	3943 ^a^							

^1^ Significances: ns: not significant; **: *p <* 0.01; ***: *p <* 0.001. The parameters in each row (for males and females) not followed with the same superscript letters are significantly different (*p* < 0.05). ^2^ Control rabbits versus obese rabbits (with hyper caloric and hyper lipidic rich diet). ^3^ BMI: Body Mass Index.

**Table 4:** Effects of the two probiotics strains supplementation on the biochemical parameters of the obesity and metabolic syndrome in rabbits including comparison of rabbit group (G), sex (S), time (T) and the interactions from experiment 2.

Parameters ^1^	Sex	TO			OB			OL			Effects ^2^						
		0	15	30	0	15	30	0	15	30	Group ^3^	Time	Sex	S × T	G × T	S × G	G × T × S
GLU g/L	M	1.83 ^a^	1.72 ^b^	1.58 ^bc^	2.01 ^a^	1.59 ^c^	1.30 ^d^	1.94 ^a^	1.73 ^bc^	1.42 ^d^	*	***	ns	ns	**	ns	ns
	F	1.95 ^a^	1.72 ^b^	1.72 ^b^	1.91 ^a^	1.59 ^c^	1.38 ^d^	2.04 ^a^	1.64 ^bc^	1.43 ^d^							
TC (g/L)	M	2.02 ^ab^	1.85 ^bc^	1.76 ^bc^	2.46 ^ab c^	1.86 ^bc^	1.47 ^d^	2.03 ^a^	1.89 ^bc^	1.51 ^d^	**	***	ns	ns	ns	***	ns
	F	2.89 ^a^	2.45 ^ab c^	2.43 ^bc^	2.57 ^ab^	1.77 ^bc^	1.59 ^d^	2.02 ^ab^	1.80 ^bc^	1.57 ^d^							
HDL (g/L)	M	0.15 ^b^	0.21 ^ab^	0.22 ^ab^	0.21 ^ab^	0.19 ^ab^	0.24 ^ab^	0.17 ^ab^	0.23 ^ab^	0.27 ^a^	ns	*	ns	ns	ns	ns	ns
	F	0.20 ^ab^	0.18 ^ab^	0.17 ^ab^	0.17 ^ab^	0.17 ^ab^	0.21 ^ab^	0.19 ^ab^	0.21 ^ab^	0.20 ^ab^							
LDL (g/L)	M	1.44 ^a^	1.37 ^bc^	1.20 ^bc^	1.55 ^a^	0.93 ^d^	0.82 ^d^	1.44 ^a^	0.98 ^d^	0.94 ^d^	**	***	ns	ns	*	ns	ns
	F	1.42 ^a^	1.25 ^bc^	1.21 ^bc^	1.50 ^a^	1.00 ^d^	0.92 ^d^	1.46 ^a^	0.84 ^d^	0.76 ^d^							
TG (g/L)	M	3.75	2.94	2.73	2.85	2.93	2.57	3.53	2.30	2.04	ns	*	ns	ns	ns	ns	ns
	F	3.08	2.67	2.83	2.83	2.43	2.03	2.83	2.70	2.22							
CREA (mg/L)	M	13.13	12.78	12.77	12.64	12.69	12.60	13.69	13.65	13.65	ns	ns	ns	ns	ns	*	ns
	F	13.00	12.97	12.91	12.85	12.76	12.47	12.18	12.18	12.09							
TP (g/L)	M	79.51	65.33	64.33	78.40	68.67	68.01	66.33	67.00	68.76	ns	ns	*	ns	ns	ns	ns
	F	68.13	88.43	88.60	84.37	82.40	66.21	89.33	81.33	69.48							
ALB (g/L)	M	47.36	42.05	42.05	44.06	43.17	43.00	38.67	38.73	38.75	ns	ns	ns	ns	ns	ns	ns
	F	41.18	42.23	42.23	36.24	40.60	39.07	46.16	45.40	45.40							
ASAT (UI/L)	M	61.90	53.33	51.14	48.75	49.93	49.13	47.53	46.67	47.90	ns	ns	ns	ns	ns	ns	ns
	F	47.10	55.33	54.00	59.13	57.83	55.40	62.73	61.30	49.83							
ALAT (UI/L)	M	47.59	46.25	47.50	52.30	42.74	41.36	45.41	42.58	42.58	ns	ns	*	ns	ns	ns	ns
	F	45.13	48.62	47.23	47.53	51.48	52.80	49.02	50.10	50.10							
ASAT/ALAT	M	1.32	1.19	1.08	1.00	1.18	1.20	1.09	1.11	1.13	ns	ns	ns	ns	ns	ns	ns
	F	1.06	1.14	1.17	1.23	1.15	1.06	1.26	1.25	1.01							
AP (UI/L)	M	102.2	104.0	103.0	107.5	100.6	99.0	99.6	100.3	101.3	ns	ns	ns	ns	ns	ns	ns
	F	102.0	105.0	105.0	104.0	104.3	105.6	108.0	106.6	104.6							

^1^ Abbreviations: GLU: Fasting blood sugar, TC: Total Cholesterol, HDL: High-Density Lipoprotein, LDL: Low-Density Lipoprotein, TG Triglycerides, CREA: Creatinine, TP: Total Protein, ALB: Albumin, ALAT: Alanine Aminotransferase, ASAT: Aspartate Aminotransferase, AP: Alkaline Phosphatase. ^2^ Significances: ns: not significant; *: *p* < 0.05; **: p < 0.01; ***: *p* < 0.001. The parameters in each row (for males and females) not followed with the same superscript letters are significantly different (*p* < 0.05). ^3^ Groups: TO: Control Obese; OB: Obese + *Bifidobacterium animalis* subsp. *lactis* BB-12 probiotic; OL: Obese + *Lactobacillus plantarum* 299v probiotic.

**Table 5:** Variations of OGTT evaluated on the rabbits over time in experiment 2 and effects of group (G), sex (S), time (T) and the interactions.

Parameters ^1^		TO			OB			OL			Effects ^2^						
		0	15	30	0	15	30	0	15	30	Group ^3^	Time	Sex	S × T	G × T	S × G	G × T × S
OGTT (T0) (g/L)	M	2.03 ^a^	1.92 ^ab^	1.92 ^ab^	1.9 ^ab^	1.16 ^de^	1.06 ^e^	1.97 ^ab^	1.56 ^bcd^	1.30 ^cde^	***	***	***	ns	***	**	ns
	F	1.81 ^ab^	1.71 ^abc^	1.62 ^abc^	1.96 ^ab^	1.13 ^de^	1.03 ^e^	1.80 ^ab^	1.11 ^e^	0.99 ^e^							
OGTT (T30) (g/L)	M	3.97	3.40	3.17	3.85	2.55	2.32	3.94	2.72	2.60	ns	ns	ns	ns	ns	ns	ns
	F	3.86	3.41	3.35	3.92	3.86	3.34	3.88	3.74	3.59							
OGTT (T60) (g/L)	M	3.17	2.90	2.59	3.27	3.07	2.94	2.87	2.63	2.52	ns	ns	ns	ns	ns	**	ns
	F	3.01	2.96	2.91	2.67	2.57	2.25	3.27	3.18	2.91							
OGTT (T90) (g/L)	M	2.46	2.08	1.92	2.72	2.47	2.31	2.36	2.23	2.14	ns	**	ns	ns	ns	ns	ns
	F	2.49	2.36	2.26	2.69	2.27	2.17	2.72	2.53	2.38							
OGTT (T120) (g/L)	M	2.19 ^ab^	1.88 ^ab^	1.78 ^ab^	2.23 ^ab^	2.04 ^ab^	1.91 ^ab^	2.09 ^ab^	1.84 ^ab^	1.71 ^ab^	*	*	ns	ns	ns	***	ns
	F	2.14 ^ab^	2.04 ^ab^	2.04 ^ab^	1.64 ^ab^	1.36 ^ab^	1.28 ^b^	2.27 ^a^	2.11 ^ab^	2.00 ^ab^							
OGTT (T180) (g/L)	M	1.20	1.73	1.49	1.11	1.00	0.98	1.28	1.07	0.96	ns	ns	**	ns	ns	ns	ns
	F	1.67	1.65	1.59	1.66	1.61	1.20	1.62	1.49	0.38							

^1^ OGTT = Oral Glucose Tolerance Test evaluated at 0, 30, 60, 90, 120 and 180 min. ^2^ Significances: ns: not significant; *: *p* < 0.05; **: *p* < 0.01; ***: *p* < 0.001. The parameters in each row (for males and females) not followed with the same superscript letters are significantly different (*p* < 0.05). ^3^ Groups: TO: Control Obese; OB: Obese + *Bifidobacterium animalis* subsp. *lactis* BB-12 probiotic; OL: Obese + *Lactobacillus plantarum* 299v probiotic.

**Table 6:** Comparison of the morphometric parameters for the effects of rabbit group, sex, time and interactions after the administration of probiotics from experiment 2.

Parameters ^1^		TO			OB			OL			Effects ^3^						
	Sex	0	15	30	0	15	30	0	15	30	Group ^2^	Time	Sex	S × T	G × T	S × G	G × T × S
Length (cm)	M	44.25	44.0	44.0	44.0	44.33	44.33	46.0	46.0	46.0	ns	ns	*	ns	ns	***	ns
	F	47.67	47.67	47.67	46.33	46.33	46.33	44.0	44.0	44.0							
Height (cm)	M	25.75	25.67	25.67	25.50	25.67	25.67	24.67	24.67	24.67	ns	ns	ns	ns	ns	ns	ns
	F	25.67	25.67	25.67	25.67	25.67	25.67	25.67	25.67	25.67							
Abdominal Circumference (AB) (cm)	M	47.00	46.33	46.33	47.0	46.67	45.33	46.67	46.33	46.0	ns	*	ns	ns	ns	ns	ns
	F	47.33	46.67	46.67	47.33	46.33	45.0	47.00	46.33	45.33							
AB/Length ratio	M	1.06	1.05	1.05	1.07	1.06	1.02	1.01	1.01	1.00	ns	ns	ns	ns	ns	**	ns
	F	1.00	0.98	0.98	1.02	1.00	0.97	1.07	1.05	1.03							
BMI ^1^ (kg/m^2^)	M	31.63	31.43	31.22	34.34	31.58	30.13	31.22	29.50	28.05	ns	*	**	ns	ns	*	ns
	F	34.13	33.45	33.38	33.33	31.50	30.15	35.84	34.40	34.05							

^1^ BMI: Body Mass Index. ^2^ Groups: TO: Control Obese; OB: Obese + *Bifidobacterium animalis* subsp. *lactis* BB-12 probiotic; OL: Obese + *Lactobacillus plantarum* 299v probiotic. ^3^ Significances: ns: not significant; *: *p* < 0.05; **: *p* < 0.01; ***: *p* < 0.001. The parameters in each row (for males and females) not followed with the same superscript letters are significantly different (*p* < 0.05).

## Data Availability

No data copyright issues.

## Supplementary Materials

The following are available online at https://www.mdpi.com/2079-7737/10/2/131/s1, Figure S1: Oral Glucose Tolerance Test (OGTT) of rabbits at D0, 8 and 14 weeks observed in the experiment 1. Figure S2: Effect of time (age) on the levels of (a) total cholesterol (TC), (b) Low-Density Lipoprotein (LDL), (c) High-Density Lipoprotein (HDL) and (d) triglycerides (TG) of rabbits at D0, 8 and 14 weeks measured in the experiment 1. Figure S3: Effect of probiotics on OGTT concentrations of the rabbits over time. Group TO: Obese witnesses (TO) rabbits used as control and without any probiotic in their feed. Group OL: Obese rabbits given 1 × 10^10^ CFU mL of *Lactobacillus plantarum* 299v. Group OB: Obese rabbits (*n* = 6) receiving 1 × 10^9^ CFU/mL of *Bifidobacterium animalis* subsp. *lactis* BB-12.
