# Supplementary material for: Study of Probiotic Effects of Bifidobacterium animalis subsp. lactis BB-12 and Lactobacillus plantarum 299v Strains on Biochemical and Morphometric Parameters of Rabbits after Obesity Induction
Source: Biology (Basel). 2021 Feb 7;10(2):131. doi: 10.3390/biology10020131 (PMC7915171; doi:10.3390/biology10020131)
Supplement: Supplementary file 1 [file biology-10-00131-s001.pdf]

biology

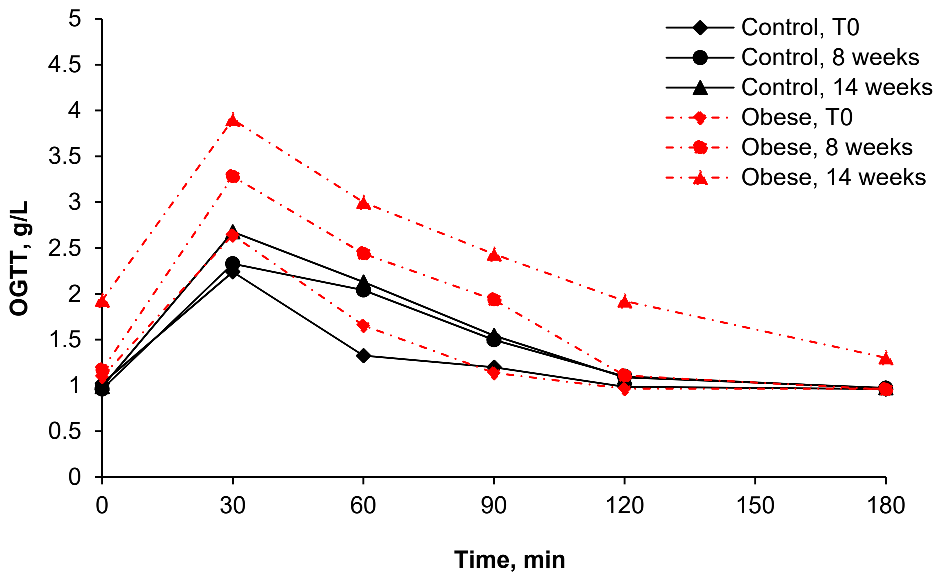

Figure S1: Oral Glucose Tolerance Test (OGTT) of rabbits at T0, 8 and 14 weeks observed in the experiment 1.

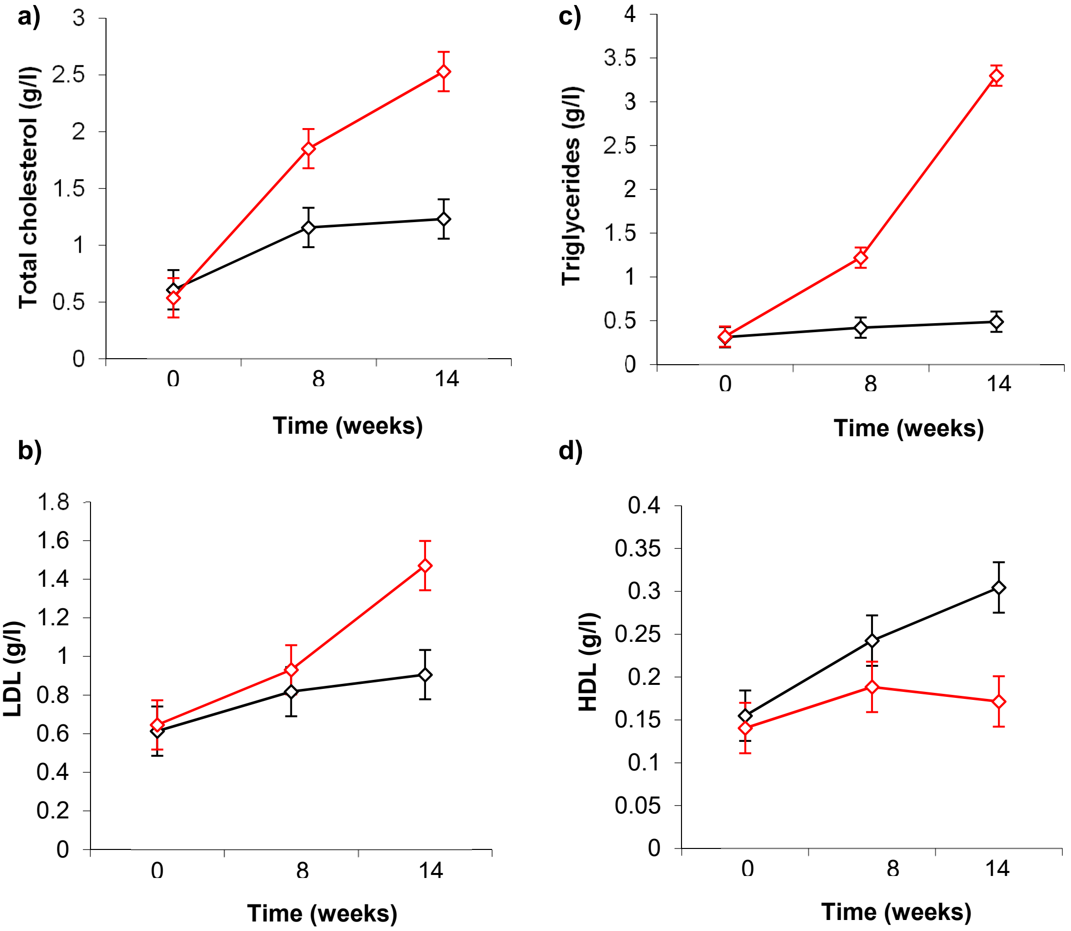

Figure S2: Effect of time (age) on the levels of (a) total cholesterol (TC), (b) Low-Density Lipoprotein (LDL), (c) triglycerides (TG) and (d) High-Density Lipoprotein (HDL) of rabbits at T0 (day 0 control), 8 and 14 weeks measured in the experiment 1.

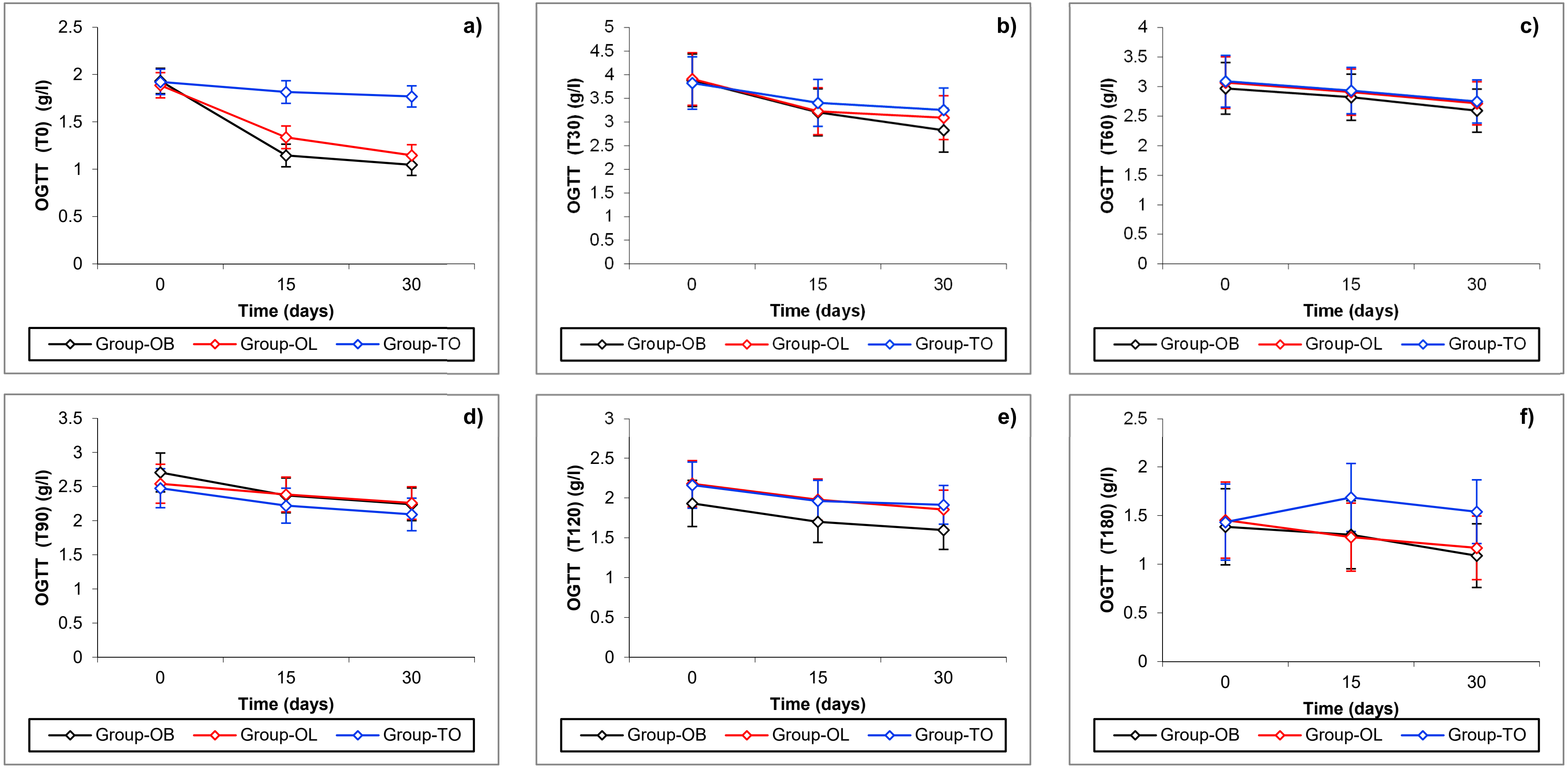

Figure S3: Effect of probiotics on OGTT concentrations of the rabbits over time. Group TO: Obese witnesses (TO) rabbits used as control and without any probiotic in their feed. Group OL: Obese rabbits given 1 × 10^{10} CFU mL of *Lactobacillus plantarum* 299v. Group OB: Obese rabbits (n = 6) receiving 1 × 10^{9} CFU / mL of *Bifidobacterium animalis* subsp. *lactis* BB-12.
